# SPAG9 expression is increased in human prostate cancer and promotes cell motility, invasion and angiogenesis *in vitro*

**DOI:** 10.3892/or.2022.8314

**Published:** 2022-04-04

**Authors:** Feifei Chen, Zheng Lu, Junpeng Deng, Xuechao Han, Jin Bai, Qinghua Liu, Yaguang Xi, Junnian Zheng

Oncol Rep 32: 2533–2540, 2014; DOI: 10.3892/or.2014.3539

Subsequently to the publication of the above article, an interested reader drew to the authors’ attention that a pair of data panels presented in each of Figs. 3 and 4 appeared to be overlapping, such that these data may have been derived from the same original sources where they were intended to have shown the results from experiments performed under different experimental conditions. The authors realised that these figures had inadvertently been assembled incorrectly; however, as they had retained their access to the raw data, the authors were able to make the appropriate corrections required for these figures.

The corrected versions of Figs. 3 and 4, showing the correct wound healing assay result for the DU1450-siSPAG9 experiment at 24 h in Fig. 3F and the correct Matrigel cell invasion assay result for PC3-siSPAG9 in Fig. 4C, are shown on the subsequent pages. Note that these errors did not adversely affect the major conclusions reported in the study. The authors all agree with these corrections and thank the Editor of *Oncology Reports* for allowing them the opportunity to publish this corrigendum. The authors also apologize for any inconvenience caused, and agree to address any additional questions regarding their results. All raw data are available from the authors upon request.

## Figures and Tables

**Figure 3.**
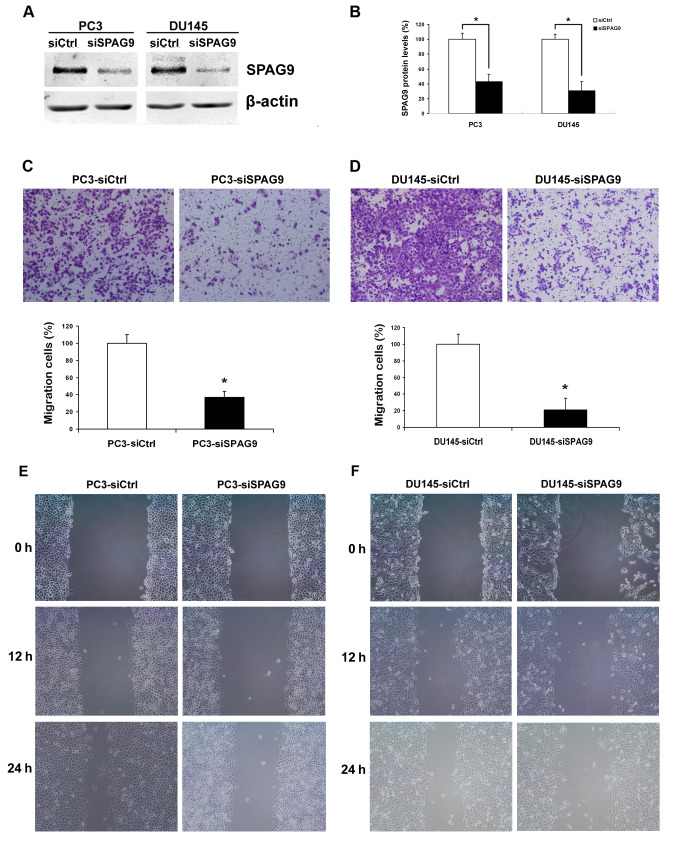
Effect of the reduction in SPAG9 expression on the abilities of cell motility *in vitro*. (A and B) Forty-eight hours after transfection, the expression of SPAG9 in the PC3 and DU145 cells was evaluated by western blotting. β-actin was used as an internal control. (*P<0.05, ANOVA) (C and D) Cell migration assay. Representative fields of migrating cells on the membrane (magnification, ×200). Average percentage of migrating cells per field. (*P<0.05, ANOVA) (E and F) Wound healing assay revealed that there was a significant delay in wound closure after knockdown of SPAG9 expression when compared with the rate of wound closure in the control group.

**Figure 4.**
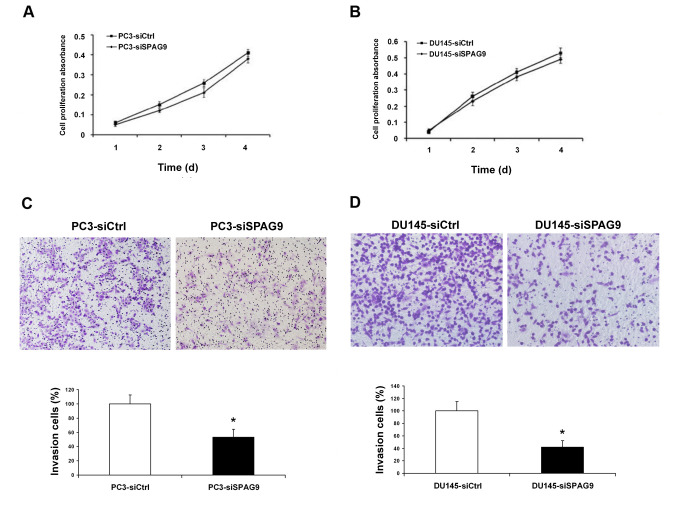
Reduction in SPAG9 expression suppresses cell invasion but not cell proliferation in prostate cancer cells. (A and B) CCK-8 cell proliferation assay was performed to detect prostate cancer cell proliferation. (C and D) Matrigel cell invasion assays. Representative images of the cells that invaded through the Matrigel following transfection with siSPAG9 or control siRNA. Representative histograms of the percentage of invaded tumor cells are displayed and the number of invaded tumor cells was quantified. *Significant difference from the controls (P<0.05, ANOVA).

